# Understanding old herbal secrets: The renaissance of traditional medicinal plants beyond the twenty classic species?

**DOI:** 10.3389/fphar.2023.1141044

**Published:** 2023-03-24

**Authors:** Marisa Milena Scherrer, Stefan Zerbe, Joshua Petelka, Ina Säumel

**Affiliations:** ^1^ Department of Geography, Humboldt-Universität zu Berlin, Berlin, Germany; ^2^ Faculty of Science and Technology, Free University of Bozen-Bolzano, Bolzano, Italy; ^3^ Conservation Administration, Regional Council Freiburg, Freiburg, Germany; ^4^ Integrative Research Institute THESys Transformation of Human-Environment-Systems, Humboldt-Universität zu Berlin, Berlin, Germany

**Keywords:** biocultural diversity, cultural erosion, cultural ecosystem services, environmental education, folk medicine, nature conservation, South Tyrol, biodiversity

## Abstract

The use of traditional medicinal plants plays an important role especially in remote rural and marginalized landscapes at different latitudes. In the development of nature conservation strategies based on local knowledge and sustainable resource management, medicinal herbs have been hypothesized to be cultural key stone species. Environmental education is a crucial driver for fostering environmental literacy and preserving local knowledge across generations. We conducted semi-structured interviews with stakeholders in the Autonomous Province of Bozen-South Tyrol (N Italy) to gain insights into, and reflections on, the cultural value of traditional medicinal plants and their interplay within the local landscape, nature conservation and their role in environmental education and knowledge transfer across generations. We also used a combination of purposive and snowball sampling to identify relevant actors. The different voices collected in the study clearly highlight the role of medicinal herbs in healthcare, for cultural identity of local communities and demonstrate a growing commercial market niche that maintains the local economy and services, including widespread offers related to environmental education, that have not, unfortunately, been used yet in the formal curricula of local schools. The latter is crucial for a holistic approach taking medicinal plants as an ideal vehicle to connect especially children with nature and history of South Tyrol, strengthening health education and overall environmental literacy, including species knowledge. However, the revival of herbal medicine and related knowledge do not prevent the continuous loss of local traditional knowledge regarding medicinal plants, recipes and use. More species and their uses are being forgotten due to superficialisation of knowledge and of mainstreaming and homogenization of the global market of herbal medicine. Safeguarding the natural and cultural treasures of South Tyrol for future generations is in the hands of the local communities.

## 1 Introduction

For many millions of people around the globe, herbal medicine is the main form of healthcare (WHO, 2019). The knowledge on medicinal plants and fungi mixed in magical rituals and customs is mostly preserved in those areas with logistically or financially difficult access to a doctor or veterinarian (Howes et al., 2020). A considerable body of literature covers interesting studies from high mountains (e.g., Tucakov, 1997; Pullaiah et al., 2017; Petelka et al., 2020), from steppe and grassland landscapes (e.g., Molares and Ladio, 2009; Pozdnyakova et al., 2022; Santoro et al., 2022) to from more or less pristine forests (e.g., Uprety et al., 2012; Rodrigues et al., 2021; Kolosova et al., 2022). Old herbal scripts in monasteries showed that herbalists have been always part of the medicinal landscape, even in easily accessible regions before the advent of conventional medicine (Green, 1989; Sabatini, 1994), but were pushed back there more quickly, despite many conventional drugs being developed based on natural remedies (Clément, 2005; Pan et al., 2014). The species name ‘*officinalis*’ (e.g., *Valeriana officinalis* L. *Calendula officinalis* L. or *Melissa officinalis* L.) literally indicates the medicinal use over many centuries as in the ‘officina’ of medieval monasteries next to herb gardens remedies were prepared (Pearn, 2010).

Herbal medicine and traditions have, to date, been discussed so far from very different perspectives. Global demand for medicinal plants and fungi has threatened certain species that are important for the human health, and contributed to the loss of biodiversity and depletion of natural resources (Howes et al., 2020). In addition to general threats to biodiversity such as both land-use intensification and land abandonment (Zerbe, 2022), and climate change (Brondizio et al., 2019), commercialization and over-exploitation of medicinal plant species can also conflict directly with species and habitat protection (Schippmann et al., 2002; Volenzo and Odiyo, 2020). Several authors have called for a new era of large-scale exploration of therapeutic candidates from nature to prepare humanity for future health challenges (Pérez-Escobar et al., 2020). In light of this global agenda, the role of local people in managing biodiversity and natural resources appears to be even more decisive. A more holistic and sustainable vision of conservation and habitat restoration is capable of incorporating cultural and socio-economic factors and making conservation efforts meaningful to local people (e.g., Volenzo and Odiyo, 2020; Zerbe, 2023). Concepts of cultural keystone species (e.g., Garibaldi and Turner, 2004; Petelka et al., 2022) or biocultural diversity (e.g., Maffi, 2018), which have built bridges from ecological to social sciences, recognize the role of species in the identity and culture of a local community, help to develop conservation strategies based on traditional ecological knowledge, and identify the local community as a key institution for sustainable resource management (Berkes et al., 2000; Volenzo and Odiyo, 2020).

In this context, the new convergence between science education and environmental education can unlock synergies between critical and integrative thinking, transformative learning and commitment to change individual and community acting as demonstrated in citizen or community science (Wals et al., 2014). These projects already connect environmental literacy with place and local identity, make the intangible global crises tangible (Wals et al., 2014; McKinley et al., 2017; Groulx et al., 2021) as well as empower people to become proactive and not give up. Without claiming that species knowledge is more developed in the animal kingdom, zoocentrism and plant blindness (Wandersee and Schussler, 1999; Frisch et al., 2010) poses a mayor problem to plant conservation (Balding and Williams, 2016). However, peer reviewed studies on environmental education using medicinal plants are scarce^1^ (though see Azevedo et al., 2015; Staniszewski et al., 2016) and, case studies in the Global South report a discrepancy between knowledge and use of medicinal plants, a decrease in the knowledge with increasing formal educational and an inter-generational erosion of knowledge (Srithi et al., 2009; de Sousa et al., 2022).

South Tyrol in the European Alps is a refugia for herbal medicine and preserved knowledge (Grabherr, 2009; Petelka et al., 2020). Traditional medicinal plants have been identified here as ideal as cultural keystone species to protect biodiversity and nature (Petelka et al., 2022). Despite the general trend of habitat loss and cultural erosion, there is growing evidence in South Tyrolian communities of renewed social and scientific interest in traditional medicinal plants and associated knowledge, which foster both biodiversity conservation and cultural identity (Pieroni and Giusti, 2009; Abbet et al., 2014; Pinela et al., 2017; Schunko et al., 2019; Petelka et al., 2020).

In this study, we explore the role of environmental education, involved stakeholders and discourses in the renaissance of traditional medicinal herbs in South Tyrol. We conducted semi-structured interviews with local experts to gain insights into, and reflections on this phenomenon, collecting their thoughts on the cultural value of traditional medicinal plants and their interplay within the local landscape, nature conservation and their role in environmental education across generations.

## 2 Materials and methods

### 2.1 Study area

The Autonomous Province of Bozen-South Tyrol, with an area of 7,400.43 km^2^ is located in the Central and Southern Alps and is the most northern province of Italy with borders with Austria in the North and to Switzerland in the West. South Tyrol is dominated by spruce and pine forests (49%), agricultural areas (mainly pastures; 23%), non-used land including rocks, water and glaciers (19%), abandoned land (6%), and settlement areas (3%). In the past few centuries, fourteen percent of meadows and pastures disappeared; grassland has been abandoned in higher altitudes, and grassland and arable land in the valleys have been converted to intensively-managed permanent crops and/or urbanized (Schirpke et al., 2020; Schirpke et al., 2021). Even so, the region has retained a long cultural history and is a hotspot of floristic diversity and of ethnopharmacological plants, in particular (Petelka et al., 2020). After centuries of belonging to the Habsburg Empire, South Tyrol became part of Italy after World War I (Steininger, 2003; Lantscher, 2008), with a governance and system of power-sharing between the three clearly separated language groups (German, Italian, Ladin) that successfully manages ethnic diversity, resolves potential conflicts and promotes interethnic cooperation (Alber, 2017; Carlà, 2015, Carlà, 2022). Ladin speakers live mainly in two valleys and Italian speakers live mainly in urban areas, especially in the South Tyrolean capital Bolzano (Carlà, 2022). Until the end of the 19th century, the economy of South Tyrol was characterized by solitary mountain farmers, smallholders and family farms. In remote areas, the use of local medicinal plants was the most important, and often the only way to treat diseases and health problems. Accordingly, unique medical customs and traditions developed, and medicinal plants became an integral part of the culture (Pickl-Herk, 1995). The attractive landscape, traditional culture and knowledge such as land-use techniques (e.g., transhumance) and costumes have made South Tyrol a well-known tourist destination (Kreisel and Reeh, 2011; Ghirardello et al., 2022).

### 2.2 Semi-structured interviews with local experts

We used qualitative, semi-structured guided interviews (Mattisek et al., 2013) with local actors to explore the role of medicinal plants in environmental education. Based on previous studies on stakeholder analysis in the region (Petelka et al., 2022), we identified people with many years of knowledge about and working experience with medicinal plants, for example, farmers, lecturers or employees in educational institutions. Interviews are a basic method for socioecological studies and do not focus on numbers, but primarily on obtaining the opinions of various stakeholders who are relevant for new insights. We define stakeholders as individuals, groups or organisations that are affected by or able to influence a particular phenomenon (i.e., medicinal plants) and are involved in decision-making processes (Reed et al., 2009). All selected interviewees are from the region and work with medicinal plants in South Tyrol, including experts with awards, recommendations, or a high profile in the region. We used a combination of purposive and snowball sampling (Bernard, 2002), explicitly seeking out actors who are active in environmental or herbal education and offer related services.

Initially, the search for interview partners was carried out by means of internet research, taking into account the knowledge already acquired. Over the course of time, the interviewed actors made further recommendations and referred to contact persons. In the first contact, by e-mail, the research objective was explained and the willingness to be interviewed was requested, assuring measures to protect confidentiality. During the interviews, notes were taken in bullet points, and in some cases individual relevant quotations were written down in full. While maintaining confidentiality, all data were anonymized in a protected space. All data were deleted after processing and analysis^2^, except the anonymized transcripts of main points (Supplementary Material). The response rate was relatively high. The majority responded in a timely manner and expressed interest in an interview. A total of nine women and five men were interviewed (Supplementary Material, anonymized data of interviewees). The age of the interviewees range between 35–70 years. Only a few young actors were available for the interview. All fourteen interviewees are German speakers, because German, besides the second official language Italian, is the lingua franca in South Tyrol.

The focus was on experiences and narratives as well as the answering of targeted questions through a guideline within the interview. The semi-structured guided interviews were conducted for about 15–45 min *via* teleconferences due to the COVID-19 restrictions. The questionnaire was sent in advance by e-mail. We extended a questionnaire previously used in the region (Petelka et al., 2022), also asking about the role of traditionally used medicinal plants for environmental education. We specifically asked the following questions: Who do you consider relevant to the topic of traditional medicinal plants in South Tyrol? (Opening question) How do you assess the interest in and influence on traditional medicinal plants? Which South Tyrolean medicinal plants do you consider to be the most important for medicinal purposes (nutritional, veterinary purposes, spiritual and cultic purposes, cosmetic and domestic)? Which South Tyrolean medicinal plants do you consider culturally most important in terms of intensity of use, ceremonies, legends, symbols, history, trade, irreplaceability? To what extent are cultural key stone species in South Tyrol formative for the conservation or restoration of ecosystems? What role do (cultural) medicinal plants play in environmental education? Do they play a role? Is there an increased focus on passing on knowledge about medicinal plants? What does the educational work look like? How relevant is the topic for children and young people and the transmission of knowledge to future generations? How can more importance be attached to knowledge about medicinal plants? What would need to be changed? Following this semi-structured set of questions, the interviews were individual and not all questions were discussed in the same depth with all interviewees (Supplementary Material). Each interviewee shed light on their own perspectives and focused on points of deeper meaning within their testimony depending on their context, experiences and interests. The insights are puzzle pieces of a larger picture that we discuss below with the literature.

We used content analysis (Mayring, 2008). At the beginning of the evaluation, the written key points, sorted according to the interviews, were imported into the software MAXQDA (VERBI Software, 2021). A keyword list was compiled on the basis of the transcripts (Supplementary Material). Codes were assigned in the software, based on the keyword list and chronologically assigned to the individual questions. Using the function “List of coded segments”, individual documents or several at the same time could then be selected and filtered according to codes such as culture, folk medicine, tradition, environmental education or awareness rising. This allowed the evaluation according to the questionnaire (Supplementary Material).

## 3 Results

### 3.1 Renaissance of traditional medicinal plants in South Tyrol

The use of medicinal plants has a long tradition in South Tyrol closely associated with herbal healing and a deep trust, even faith, in the power of plants (I4). *Interest seems to grow from year to year. At a time when people’s longing for peace, relaxation and nature is growing, there is a renaissance of medicinal herbs all over Europe* (I2^3^). Two-thirds of all interviewees reported an increasing interest in medicinal plants, especially in wild herbs, in South Tyrol in recent years. This revival can build on a knowledge base that continues to exist after interest waned in the 1980s and 1990s (I4). These are primarily preventive applications to maintain health, sometimes also to relieve pain, although, as one interviewee mentions, the profession of alternative practitioner, for example, with which such applications are generally associated, does not exist in Italy (I3).

*The return of people to nature and regionality gives confidence for the future. Even earlier times can be better understood through traditions* (I7). Basically, the interest is stronger among women than among men. Women express their interest in healing knowledge mainly through their pronounced interest in sustainability and environmental protection, which is often aroused during child-bearing phases. Awareness of sustainability also includes the use of regional and local plants. While in the past the majority of those interested were in the middle age group of 40 and older, interest among the youth has also increased strongly in the last two to 3 years, with many of them coming into touch with medicinal plants *via* their parents aged around 50 (I12). In addition, the younger generation is now mainly interested in the background and causes of the use of plants with medicinal effects. The older generation has questioned negative consequences or side effects less often than the youth and some misinformation has also been spread.

*In the past*, it was basically a lack of income and access to conventional medicine that led to an increased use of plants for cold symptoms or other diseases (I3). The progress of conventional medicine, the employment of women as well as the introduction of compulsory education initially led to the suppression of herbalism (I3). A quarter of the interviewees stated that a lot of herbal knowledge had been lost due to orthodox medicine. The effectiveness of antibiotics was considered comparatively higher compared to herbs and the word ‘health’ was only allowed to be used by doctors (I3). However, there is no complete displacement of knowledge by orthodox medicine. The interviewees are open to the use of synthetic medicines. Conventional medicine should not be seen as alternative, but as complementary to herbal medicine, since methods of application are not mutually exclusive, but complementary (I3).

Most interviewees highlighted that the original traditional knowledge and interest is not congruent with the current rediscovered knowledge on herbal medicine. The latter is described in many interviews as hype, trend or as ‘esotericism’ and as opposed to a well-founded engagement with medicinal plants by a selected clientele (I5). The traditional empirical medicine is only present in traces and “the original heterogeneity of herbal medicine is no longer given” (I9). *In former times, the occurrence and use of medicinal plants differed from valley to valley within South Tyrol.* Today, the occurrence of species varies little between, for example, Central Germany and Italy, since comparable knowledge is available through herbal books and educational offers and, in addition, further challenges such as climatic changes are added and limit the occurrence of the plants. *The active use and application of important medicinal plants has been reduced from about 400 to 20 species—so-called “mainstream plants”* (I9).

### 3.2 Use of traditional medicinal plants in South Tyrol

*There is not THE most important one, but certainly several that are used* (I11). *It is difficult to break it down to a few plants* (I12). *Arnica montana* L. is mentioned in 8 of the 14 interviews as one of the most effective medicinal plants. In more than half of the interviews, *Arnica montana*, *Peucedanum ostruthium* (L.) W. D. J. Koch, *Calendula officinalis*, *Achillea millefolium* L. and *Urtica dioica* L. are mentioned several times. *Achillea millefolium, Urtica dioica*, *Hypericum perforatum* L. *Arnica montana*, *Verbascum densiflorum* Bertol. and *Thymus pulegioides* L. are native. *Calendula* is also one of the well-known pioneer plants, as is *Matricaria chamomilla* L. and the queen of native wild plants *Urtica dioica* (I1). *Taraxacum* spec. plays a major role in everyday nutrition as it is significant for cleansing and as a liver plant. *Achillea millefolium* is considered as a panacea and is mentioned in half of the interviews.

Almost half of the interviewees differentiated between plants of empirical medicine and those of modern herbal medicine. *Achillea millefolium* and *Alchemilla xanthochlora* Rothm. are considered typical plants of empirical medicine, but not necessarily used in modern herbalism. In comparison, *Arnica montana*, *Pinus mugo* Turra, *Juniperus communis* L. subsp. *communis* and *Symphytum officinale* L. are considered plants of modern herbalism. Others differentiated according to the occurrence of the plants in the south, north or mountain regions. *Sambucus nigra* L. is specifically referred to in the course of three interviews: *You have to take your hat off to elder and you have to kneel down to juniper* (I3). This was an important component of the farmer’s pharmacy, especially in times past, although no flowers were used. It is precisely because of its adaptability to very different conditions that elder is a strong medicinal plant. Berries, blossoms, and in earlier times also the bark and roots of the plant were used.

In total, there are 45 growers in the region who cultivate, dry, process, and market medicinal plants and wild herbs (I8). Climatic conditions are crucial and the occurrence of the plants varies across the region. For instance, plants that like harsh climates usually grow better in South Tyrol. The general use of the plants is shown by naturally obtained ointments in the medicine cabinet, such as the production of larch resin ointment, which helps against abscesses, splinters, and tension, or bronchial, calendula or arnica ointment. The lists or mentions of the most important plants vary. One interview partner reported that the German-speaking population is ahead of the Italian-speaking population in terms of knowledge and applications of medicinal plants whereas wild herb cooking including the use of *Taraxacum* spec. or *Humulus lupulus* L. is mainly influenced by Italian and offers learning opportunities (I3). The general use is individual and need-oriented and shaped by family backgrounds and stories. The valuable medicinal plants, especially their powers are underestimated in most cases (I13).

### 3.3 Cultural value of traditional medicinal plants in South Tyrol

*Culture is a mixture of old and new knowledge that is passed on to the next generations, with new knowledge building on old knowledge* (I14). *Taraxacum* spec. *Urtica dioica*, *Achillea millefolium*, *Alchemilla xanthochlora*, *Valeriana officinalis, Sambucus nigra, Rosa canina* L. and *Tilia cordata* L. have been mentioned with characteristics of cultural keystone species. Many medicinal herbs such as *Calendula officinalis*, *Melissa officinalis*, and *Mentha* spec. are now mainly cultivated (I4). With the transition from the use of wild herbs to cultivated medicinal herbs, a lot of locally anchored knowledge has been lost, knowledge linked to customs and traditional celebrations or passed down in Ladin or German legends or fairy tales. Few customs persisted over time, and they also vary from place to place, and family.

About a quarter of the interviewees reported active customs that have been applied across generations (I2- I4, I9, I12). Gathering certain plants and tying them, or making stitched baskets, is still present. Some ceremonies such as the burning of herbs, such as *Juniperus communis* subsp. *communis* are mentioned by every second interviewee. However, actual consecration only takes place in the church, although it was reported that people who know herbs still do so privately. The so-called “women’s herbs” were smoked so that the woman and her child could be bedded on fragrant herbs. The herb consecrations on fifteen of August (the Assumption of Mary) are still practiced. The herb bouquet (“Kräuerbuschen Neunerlei”; I11, I9) consisting of *Verbascum densiflorum*, *Hypericum perforatum*, *Centaurium* spec., *Achillea millefolium*, *Matricaria chamomilla*, *Artemisia absinthium* L., *Valeriana officinali*s, *Mentha* spec., and *Arnica montana* collected during the ‘womans days’ (Frauentage) between 15th August and 8th September is carried into the church for blessing. *Salix caprea* L., catkins were used to prevent thunderstorms or are consumed in spring to be protected against sore throats and colds all year round (I9). Another vivid custom is the Rauhnächte (‘Smoky Nights’ between Christmas and Epiphany) when herbs, especially *Achillea millefolium* the “master of all roots” (Meisterwurz) are smoked. The customs are not exclusively linked to ecclesiastical traditions, rather seasonal festivals are the origin for religious celebrations: *Every church festival is a nature festival* (I12). Christmas, for example, as the birth of light, is seen synonymously with the days becoming longer and brighter again. At the same time, plants are also smoked. Easter is seen as the resurrection of nature, thanksgiving for the herbal harvest. Customs, which were much more prominent in the past, are not only linked to church holidays, however; legends or fairy tales are also an integral part. In the past, there were many stories that anchored knowledge more firmly. *Customs [* … *] must not be forgotten!* (I12).

### 3.4 Traditional medicinal plants and landscape

The opinions of the respondents on the connection between traditional medicinal plants and nature conservation differ more widely than on other questions. *The collection and use of medicinal plants is closely linked to the careful handling of landscape* (I9). Awareness leads decisively to the correct handling of plants and connects to nature conservation, while *setting up prohibitions is not conducive* (I4). Others see the use of medicinal plants to be in conflict with ecosystem preservation. Medicinal plants such as *Anagallis* spec., *Centaurium* spec. or *Arnica montana* are already threatened with extinction or have already disappeared due to general eutrophication within the landscape (I9). In order to effectively counteract the effects of intensified agriculture and over-exploitation, nature conservation needs priority over the dubious use of medicinal plants, whose effectiveness has rarely been proven (I5).

**FIGURE 1:**
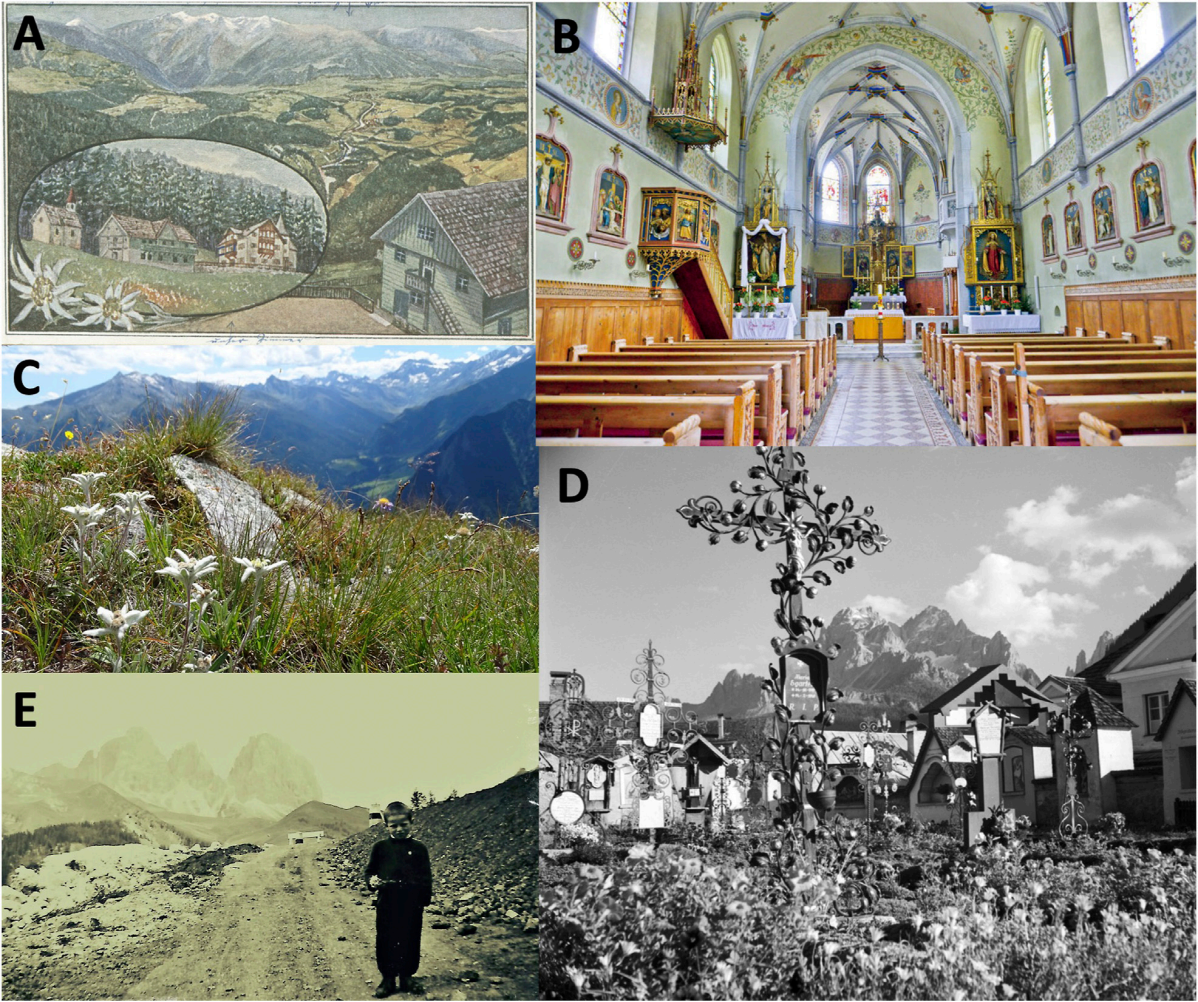
Some impressions on traditional medicinal plants in South Tyrolean landscapes. **(A)** Postcard Bad Ramwald with *Leontopodium nivale* subsp. alpinum Cass. near St. Lorenzen in the Pustertal, South Tyrol (app. 1314m), 1910, Collection Monika Weissteiner, Municipal Archives Brunico. **(B)** Inside view of the parish church/Chiesa parrocchiale, Graun-Pedross, South Tyrol (app. 1520m). The neo-Gothic church in Pedross was built from 1908 to 1912. The patron saint of the church is St. Martin. On the ceiling of the whole church are depicted various herbs, flowers and medicinal plants. Photographer: Walter Rampl **(C)** *Leontopodium nivale* in the Passeier Valley (app. 2100m). Photographer: Uwe Häntsch (Flickr). **(D)** The cemetery in Sexten/Sesto, Pustertal, South Tyrol, (app. 1310m). Old metal grave crosses have often ornamental motifs such as leaves, flowers and fruits. The flowers of *Calendula officinalis* decorate traditionally the graves and sets a counterpoint to death with its lively growth. This use has also given it the name “flower of the dead”. 1965, Collection Foto Baptist, Tyrolean Archive for Photographic Documentation and Art. **(E)** Child selling *Leontopodium nivale* to tourists in South Tyrol around 1900. Historical photo, photographer unknown. All sources are CC BY 4.0.

The current revival is also accompanied by the threat of overharvesting wild plants. *Arnica montana*, for example, can only be harvested for personal use according to very specific regulations. The charismatic *Leontopodium nivale* is protected and cannot be harvested and used, at all (I9; Figure 1). An adapted regulation for these plants, such as a ban on picking or uprooting in protected areas, is needed (I10). The rule of 10 applies to collecting. 10 plants may be collected each day, as long as they are not protected. This possibility is limited by the rule of 15 ‘freely collectable plants’, which includes plants such as *Achillea millefoliu*m, *Urtica dioica* or *Sambucus nigra*, which typically occur especially in alpine mountain altitudes, are examples for overuse. *Arnica montana* is a protected species and may not be harvested. Besides natural contamination of the plant by boring flies, the plant is also considered an example of (over)exploitation, as some outsiders and non-locals travel to South Tyrol to harvest *Arnica montana* in order to make profit with it on markets in southern Italy. The local population reacts sensitively to the endangerment of important “top medicinal plants” such as *Arnica montana* (I4). The Nature Conservation Act protects some plants, such as *Arnica montana*, in order to avoid over-exploitation. This species is designated as a Fauna-Flora-Habitat species under the European Nature Conservation Directive. Normally, a collection permit with designated purpose for plants is required but often disregarded (I5), causing considerable damage to nature.

In summary, about three-quarters of the interviewees doubt that the cultural value of medicinal plants can be combined with the idea of nature conservation, with the increasing interest in recent years being seen particularly ambivalently. In some cases, however, communities are already actively involved and the special importance of a village community for such projects was emphasized several times (I4). The aim is to create understanding among the population about why money has to flow into nature conservation (I4), but *it is not the amount that matters but the harmony* (I3). Environmental education can greatly contribute to this.

### 3.5 Traditional medicinal plants and environmental education

Mindfulness towards nature and the conscious and sustainable collection of plants must be learned: *Whoever knows nature, loves and appreciates nature* (I12). *Even in the past, there were always herb men and women in the region. Nobody knew exactly what they did with their personal healing powers and ointments* (I13). It is reported that there have been many changes in the region in the last 10 years regarding the different training centers. Fifteen years ago, there was only a very limited provision of courses, further and advanced training on medicinal plants (I12). In particular, there are educational institutions such as the Kloster Neustift near the City of Bressanone, which was mentioned most frequently, Schloss Goldrain in the Vinschgau Valley or the Kräuterakademie^4^. These ensure that a large number of interested people can be trained as herbal experts and passed knowledge on to interested people, who are diverse, both in age and profession, and ranging from academics, farmers, and educators to people in the caring professions. Most of them use the knowledge from these courses for private purposes, only a few for professional purposes (I12).

Herbal experts focus on getting to know nature with over 400 different alpine plants, garden, and wild herbs. It was emphasized, that the aim is to get to know nature without any directly associated value judgement as poisonous plants have a similar value as medicinal herbs. Two-thirds of the herbal lectures take place outdoors at different locations and one-third indoors, where tinctures and ointments are made or customs of incense traditions are transmitted (I12). With its herb cultivation, the Agricultural Research Centre Laimburg^5^ in South Tyrol is also an important educational location for farmers and interested people. Two interviewees mentioned that the Winter School Ulten^6^ offers a 3-years herbal pedagogue training. The “Kräuterkraft" (herb power) blog^7^ of the South Tyrolean herb women provides information on various topics, refers to publications, and offers networking possibilities especially between herb women. *In the meantime, the catering industry has also strongly gotten on the track* (I12). Whereas 30 years ago, there was no interest in herbal walks or similar offers, today practically every larger hotel has such offers in its programme, for which many professionals are needed (I12).

The educational provision around medical herbs changes constantly (I1, I7), and ranges from herb walks, cooking events to the active involvement of interested people in harvesting or processing the plants, according to about half of the interviewees. The goal, especially for tourism, is to create offerings that are as broad as possible, even for the uninformed, in order to expand general knowledge and herbal literacy (I7). In addition to the larger institutions, there are also individual persons who have a decisive influence on the education offered and drive the development of knowledge. These include various herbalists who offer specific courses, but also, for example, pharmacists or speakers who are generally known in the region. The Kräuterakademie of the FNL (Freunde naturgemäßer Lebensweise; Friends of Natural Living^8^) also offers courses and there are health consultations in the field of alternative healing methods.

It is reported that there have been many changes in the region regarding the different training places in the last 10 years. 15 years ago, there was only a very limited offer of courses, further and advanced training on medicinal plants (I12). In particular, educational institutions such as the Kloster Neustift, which was mentioned most frequently, Schloss Goldrain in the Vinschgau Valley or the Herbal Academy ensure that a large number of interested people can be trained as herbal experts and that the knowledge is passed on to interested people. Those interested in such training courses are diverse, both in age and profession, and range from academics, farmers and educators to people in the caring professions. Most of them use the knowledge from these courses mainly for private purposes, only a small part uses it for professional purposes (I12).

*Herbal education is knowledge transfer with fun, but also being active yourself and learning something* (I1). That it is very important to create awareness, which can be promoted through an emotional connection to nature, is often been mentioned. Environmental education plays a key role in this, whether in childhood, adolescence or adulthood, and also plays a role in society in South Tyrol.

After considering medicinal plants in the context of restoration ecology and the practice of ecosystem restoration, the following questions arise for environmental education: How really relevant is knowledge about medicinal plants actually? What is the role of environmental education and the transmission of knowledge to future generations? What environmental education institutions exist in the region and how can more importance be attached to the topic? These questions were asked at the end of the interviews in order to focus more on the future engagement with the topic.

Many associate environmental education and medicinal plants with guided tours through herb gardens, with recipes, processing of herbs in the kitchen for adults and children, as well as special children’s afternoons or courses. In South Tyrol, a well-positioned educational landscape has been built up over the last 10 years with many free offers, also thanks to a financial support from the province. Many herbal educators communicate offerings and the importance of using herbs to maintain health and enjoy life (I3). Most of the offers are focused on adult education (I1). There are guided tours all year round, in spring especially on wild herbs and medicinal plants. In addition, there are special offerings for schools and kindergartens, for example, from nature conservation associations that organise herb treasure hunts or information days on the topic of forests and meadows, or orchard meadow projects organised by the State Office for Agriculture (LFL), especially in autumn. The Laimburg technical college also offers herb courses, as do the adult education centers in the province, the Kloster Neustift and various educational houses. Some youth centers or organizations have a garden that is open to the public and offer projects in summer (I3). Such activities are very significant. The acquisition of knowledge takes place over many years and is a long process. Raising awareness from person to person is most important for this and much more difficult to achieve in school than in the family or a private environment.

Herbal experts and educators are central to the external transfer of knowledge. The offerings of environmental education and the knowledge about them vary greatly depending on the context in which the respective actors are active, because every education across South Tyrol varies and has a variety of focus. Overall, the aim is to reach all sections of the population, including children, young people, adults, and senior citizens.

*Knowledge on flora, i.e., real knowledge on species, needs to be deepened because this is hardly promoted and is rapidly being lost due to dwindling publications* (I5). For almost all experts, medicinal plants do not play a designated role in environmental education of schools, but rather appear in the context of general botany, alongside wild herbs, for example,. Others say that specific medicinal plants play a role in environmental education in private and family contexts. The importance of medicinal plants is accelerated by the media interest in the progress of bee mortality (I3). Experiencing the diversity of nature through the example of medicinal plants and their use for one’s own wellbeing also helps to influence one’s behavior towards nature and to approaching it with respect and appreciation. In addition, responsibility is assumed, so also strengthening cultural identity (I11). Cultural linkage is omnipresent. There are many books, stories, legends, and sagas for every single medicinal plant presented. Medicinal plants thus serve as a very good access point for environmental education among children.

As far as basic knowledge is concerned, there is little difference between urban and rural areas, most children know little about plants (I9). Appropriate offers must be differentiated (I2). Stories that are exciting for children are central for environmental education, because stories are remembered and facilitate the personal linking of theory and practice (I9). Particularly at primary school age, children are very interested, depending on their parents’ home, while already at adolescence different interests and the question of motivation to deal with certain topics are characteristic. In order to create positive memories that lead to awareness-raising for the future, offerings must therefore be designed primarily for children and young people. Practical work should have top priority, on the basis of the principle: *What I know, I can only protect and (not destroy)* (I1).

## 4 Discussion

The great majority of interviewees highlighted the renaissance of traditional medicinal plants after a decline in the 1980s and 1990s (e.g., I1-5, I7 -I4). This supports results of previous studies in the region (Grabherr et al., 2009; Petelka et al., 2020; Petelka et al., 2022; Cavalloro et al., 2022). Most interviewees emphasise the great treasure of traditional medicinal plants and underline their potential to enhance regional sustainability, protect biodiversity and to face current global challenges such as ecosystem degradation, environmental and health crises or cultural desintegration (Anyinam, 1995; Alves and Rosa, 2007).

Traditional medicinal plants are deeply rooted in South Tyrolian history. All respondents refer to an undefined past, to earlier or past times (e.g., *in the past, medicinal herbs were irreplaceable in daily life, ceremonies and legends;* I4, I9). Until the late 19th century, the population of South Tyrol was dominated by solitary mountain farmers and smallholders in remote family farms. The knowledge on and use of local medicinal plants was essential for survival. Numerous medical customs and traditions developed around medicinal plants, which were passed on mostly orally on from generation to generation (Pickl-Herk, 1995; Schunko et al., 2019). While religious-demonological and magical-alchemical rituals were a part of the daily life in the past, the healing applications dominate use today (ZDN, 2001). However, interviewees often mentioned a still strong linkage of medicinal herbs to secular or religious costumes and to food (e.g., Rauhnächte, woman herbs, “Neunerlei” bunch; I1- I4, I7-I9, I11-I14). Herb picking was determined by rituals (I8-9, I11, I13-14) as reported before by other studies (Łuczaj, 2011; Quave et al., 2012; Nedelcheva and Draganov 2014; Łuczaj et al., 2022). Similarly, ritual botanicals were often mentioned (I1, I3, I9, I13) protecting indirectly health from ‘bad spirits’ or ‘lightning’ combined with spells and beliefs (*A horse chestnut in your pocket protects you from bad energies* (I3); see also Pieroni and Giusti 2002; Napoli 2008; Nedelcheva and Draganov 2014; Sõukand and Pieroni 2016).

Folk medicine in South Tyrol is still practiced by people from very different groups (I2-3, I9, I11, I13). Although in the past “*herb men and women*” were the main agent of the medicinal landscape (I13), over the last few centuries folk practices have become more widespread and are no longer in the hands of special knowledge people (healers) but of the elderly (Pieroni, 2000; but see Napoli, 2008). Interviewees highlighted that the interested public is getting younger in South Tyrol (I1, I4), today it is mainly women over 40 who work with medicinal herbs in South Tyrol. These women often get in touch with medicinal herbs in their fertility phase (I12). Thus, medicinal herbs became an integral part of public health education (*most participants of the herb courses gather knowledge for their personal life and not for professional purposes;* I4, I7-I9, I12). The examples given by interviewees underline that herbal medicine is associated with either mild diseases or are starting treatment before applying conventional medicine (e.g., I3; Welz et al., 2018). In the mission documents of the South Tyrolian Museum of Pharmacy in Brixen/Bressanone, the tradition of using medicinal herbs is only mentioned as a minor matter, the museum has a herb garden, herb books in the library, a herbarium from 1,653 is preserved and the preparation of so-called ‘home remedies’ such as teas are discussed^9^.

Medicinal plants play a prominent role in the South Tyrolian environmental education for the general public inviting local people and visitors to reflect on the past, present and future as individuals and as community (I1-I4, I7). A wide portfolio of activities for trainings and knowledge exchange on medicinal herbs is offered from different institutions ranging from herb excursions, alpine forest or hey bathing to learning about the habitat of medicinal plants, use and remedy preparation and guided visits about farming in herb gardens. Educational and so called ‘social farming’ activities, provided often by women farmers, are small additional incomes for the farm and, most importantly, transmit and help to transform the local (agri)-cultural values in South Tyrol (Gramm et al., 2019). Herb educators offer seasonal schools (*with herbs healthy through the year*) and afternoon activities in schools (*we herb bitches*). The target audience ranges from kindergartens, schools to elderly people and from academics, care professions to farmers (I12). However, to unlock the whole potential also for the formal school curricula, more support from politics and state educational institutions is needed (I3, I13). About 80% of our interviewees do not have sufficient knowledge about environmental education in schools. Currently, neither the official South Tyrolian framework of the school curricula of the primary, secondary and high schools nor the environmental education offers mention medicinal herbs or traditional knowledge on herbal medicine (APB, 2021a; APB, 2021b; APB, 2022). However, some teachers do cover this topic in their classes. It is critical to integrate environmental education on medicinal plants into health education in schools. Medicinal plants serve well as a starting point for the possibility of self-help for mild or moderate complaints. Moreover, this can address the phenomenon that most children and young people have very limited connection to nature in both rural and urban settings and lack opportunities to learn about South Tyrolean nature and its medicinal plants. Many synergies with existing environmental education programme can be used by a proper training of teachers on the cosmos of medicinal plants and biodiversity, but this is also a question of funding and is currently limited by political will and funding (I12). Recently, the repetition of a survey on children’s experience of nature previously conducted over a century ago, allowed a comparison of children’s nature experience after more than four generations (Novotný et al., 2021). The results suggest that the nature experience of contemporary children is significantly higher than that of children in 1,900 (the photograph of the child in Figure 1E is from 1,919). This can be attributed in part to mediated experiences that were not available over 100 years ago. The contemporary results also show children living in a village reported higher levels of nature experience than children living in cities and the higher level of nature experience among children today compared to children in 1900 (Novotný et al., 2021).

Beyond the aforementioned lack of research on integrating medicinal herbs into environmental education in Central Europe (except Malatinszky, 2007; Azevedo et al., 2015), we identified interesting experiences in the Latin American dialogues on critical environmental pedagogy after Paulo Freire and Enrique Leff that address the intertwined environmental and educational crises (e.g., de Oliveira Martins and Ramos Araujo, 2021) and successfully integrate traditional knowledge of medicinal plants into rural school biology curricula in southern Brazil (Vinholi Júnior and Gonçalves de Azevedo, 2020). There is evidence that this practice reduces the distance both between school and regional culture and between school and everyday life, preserving cultural identity, traditional knowledge of medicinal plants, their meaning, symbolism, magic and reality (Vinholi Júnior and Vargas, 2014; Vinholi Júnior and Vargas, 2015; Souza Silva and Santos Baptista, 2018; Sousa et al., 2022; Ursi et al., 2018). In addition, the use of medicinal plants as an “educational tool” increases environmental literacy, including species knowledge, and uses the school’s immediate environment as a triple didactic resource: First, to explore and discover the world through observation and contact; second, as a starting point for the development of integrated learning projects; and third, as a means to transform desires and feelings into active proposals, opportunities to change reality, and meaningful learning as a basis for emancipated citizenship (Säumel et al., 2017; Vinholi Júnior and Gonçalves de Azevedo, 2020). The integration of medicinal plants into textbooks and curricula can increase both the quantity of knowledge offered about plants (Amprazis and Papadopoulou, 2018; Amprazis and Papadopoulou, 2020), as well as the quality of knowledge through their direct link to local history, food, medicinal and other uses (Halberstein, 2005). Creating an approach to the plant world from these local everyday perspectives strengthens place-based education and communities (Sobel, 2004; Bertling, 2018; Amprazis and Papadopoulou, 2020). Knowledge linked to where people live is authentic, more experiential and more sustainable (Amprazis and Papadopoulou, 2020).

Even the tourist industry is using the medicinal herbs culture as unique selling point for promotion campaigns. Medicinal plants have thus also become a part of a growing commercial market niche (Petelka et al., 2020; Petelka et al., 2022), linking touristic offerings with environmental education (e.g., herbal excursions, cooking or remedies preparation seminars). Hotels give their rooms name of medicinal herbs. The key to success is that medicinal plants appeal to all the senses, they can be smelled and tasted and they are associated with fairy tales and legends (I1, I9, I14). Using vernacular names in environmental education makes it easier to establish a connection to the medicinal plants (I14). Cultural importance is also highlighted by the great variety of vernacular names that can differ from valley to valley (Nedelcheva and Draganov 2014; Petelka et al., 2020). However, the established education activities have not been subject of (published) research so it remains unknown whether the knowledge shared in all these activities is also used in the daily life of participants (I2). Moreover, there is ambivalent interaction between cultural practice and the tourism, as has already been critically discussed with regard to the role of minority languages (Ladin in case of some South Tyrolean valleys; Lonardi and Unterpertinger, 2022) or festivals associated to South Tyrolean transhumance (Ghirardello et al., 2022). While these have been marketed for tourism, enhance awareness and can provide an additional income for the local community, they also detach customs from original practices, even alienate them from the local actors’ day-to-day lives by “spectacularizing” such folklore elements and thus ultimately eroding a distinct intangible heritage tradition.

That acquisition of knowledge lasts over many years is highlighted several times. There are widespread concerns about the sustainability of the revival of medicinal herb knowledge as the active use of medicinal plants has been reduced from about 400 to around 20 mainstream species (e.g., *Arnica montana, Hypericum perforatum, Urtica dioica, Plantago lanceolata* L.*, Alchemilla xanthochlora, Achillea millefolium, Juniperus communis* subsp. *communis, Matricaria chamomilla, Betula pendula* Roth.). The most often mentioned species are in line with those mentioned in similar studies in South Tyrol (Petelka et al., 2022) or in the neighbouring Province of Trento (Cavalloro et al., 2022) and, for example, in the Transylvanian or Balkan region (Živković et al., 2020; Quave and Pieroni 2014; Sõukand and Pieroni, 2016). According to the interviewees, South Tyrol is witnessing the mainstreamed popularization of certain medicinal plants (“*the glorious 20*”; e.g., I1, I9) and its marketing. This is an European trend (Quave et al., 2012). Despite of the revival of herbal medicine, local traditional knowledge regarding medicinal plants, recipes and use is continuously declining, and species are being forgotten, although researchers aim to document, preserve, and compare data concerning these unique local ethnomedical practices (e.g., Łuczaj, 2011; Quave et al., 2012; Sõukand and Pieroni, 2016; Kolosova et al., 2022). In fortunate cases when historical data are available, as in case of Łuczaj (2011) from the Western Carpathians, the ritual bouquets of the end of the 19th century could be compared with those of the beginning of the 21st century, revealing changes in the species spectrum, which also testify to the changes in habitats. The homogenization and focus on few mainstream plants are driven by different agents: 1) species recognition skills continue to decline and gathered herbal knowledge remains superficial and does not cover the great variety of species and uses (I3, I5, I9). 2) The current (also very commercialized) ‘hype’ (I5, I9, I12) is focussing on these mainstream (cosmopolitan) plants accompanied with some newly introduced species from the global village that became part of the current pharmacopaia (e.g., *Eucalyptus*, ginger). 3). Some species disappeared together with the diseases (e.g., the *Scrophularia* species; Shikov et al., 2014; Sõukand and Pieroni, 2016). 4) Theoretical knowledge of an increasingly urban society, buying herbs in pharmacies, herbal shops or supermarkets is not related to practical experiences in and knowledge on the habitat of the medicinal herb (I3). So-called “city pharmacopoeias” result from complex hybridisation processes (Ladio and Acosta, 2019; Dutta et al., 2021). It remains a major challenge, not only for ethnobotanists, to distinguish between traditional local plant use, its innovation and transformation in the context of these amalgamation processes in the global village (Leonti, 2011; Quave et al., 2012). These transformations of European ethnopharmacopeias have been exemplified as a viral narrative promoted a forgotten herbal tea as a “tradition” (Prakofjewa et al., 2020; Sõukand et al., 2020).

Interviewees shared *pros and cons* regarding the linkages of nature conservation and traditional medicinal plants. *Only what you know you will protect* (I1, I4, I9). All interviewees appreciate habitat protection measures and restrictions for gathering herbs in order to avoid overuse especially of those species that cannot be cultivated. *We need to learn mindfulness with nature and collect in such a way that one does not notice that it has been collected* (I12). Although, commercial collecting was mentioned critically by some interviewees (I4), and interest in wild plant commercialisation is increasing in South Tyrol (Schunko et al., 2019; Petelka et al., 2022), today collecting wild plants in Tyrol is mostly a leisure activity, related to traditions and not a source of income as in other parts of the world (Grasser et al., 2012; Schunko et al., 2012; Schunko et al., 2015). Wild plant gathering by local community members and combined with social events where generations can meet, exchange knowledge and strengthen local identity such as the mountain tea afternoons reported by Grasser et al. (2012) for a neighbouring region is a form of intangible cultural heritage. Conservation with local people (Petelka et al., 2022) seems more sustainable and successful in the longer term than, for example, working with highly engineered *ex-situ* seed banks, some of which are located in completely different countries or even continents (e.g., Millennium Seed Bank or Valbard Global Seed Vault). It is crucial to avoid the loss of the priceless agronomical, biological and cultural diversity connected to these genetic resources as demonstrated for old crop varieties from Northern Italy including Trentino-Alto Adige (Canella et al., 2022). Local communities are crucial in stopping the erosion of traditional knowledge and practices of sustainable resource management (Berkes et al., 2000; Volenzo and Odiyo, 2020), beyond the preservation of seeds in distant bunkers.

Several interviewees highlighted, that the rediscovered knowledge on herbal medicine is different from the original traditional knowledge, which differed from valley to valley within South Tyrol and even from family to family and therefore concluded that “*traditional empirical medicine remains only in traces”*. The same was reported for the customs associated with medicinal herbs and *vice versa*. In the past, knowledge was gained through independent practical experience in daily life from childhood, during family meals, walks through the meadows with the grandparents or in the garden and was linked to memories of childhood, beloved relatives and magic fairy tales. “*Every medicinal plant has an exciting story, not only for children* (I9).*”* Today much knowledge on medicinal plants is acquired primarily through books or is sometimes ‘obtained’. “*The experience-based knowledge of a 90-year-old is incomparable with knowledge of a 30-year-old*” who read information from books or internet (I5).

However, some interviewees still underline that the general use is individual and need oriented and shaped by family background and stories. Family traditions of using medicinal herbs are often mentioned (I1, I3-4; Ugulu and Aydin, 2011; Bruschi et al., 2019) and even more important than consulting medicinal experts (Welz et al., 2018). Although, there are many efforts to formalize the use of traditional medicine as complementary to conventional medicine (Quave et al., 2012; WHO, 2019), our interviews reveal the complicated position of traditional medicinal plants between conventional medicine, esoteric hype, empirical medicine, and modern herbalism among others.

## 5 Conclusion

The long herbal history of Europe is exemplified by the voices of practitioners of South Tyrol collected in this study illustrating not only the potential of these unique plant-human relations and interactions but also the danger that traditional knowledge about medicinal plants, their habitats, and numerous species themselves are being lost. Herb educators play a crucial role in preserving these knowledge and customs passing on practical knowledge beyond the numerous herbal books to future generations, so meet an extremely open and interested local community that is looking for a sustainable approach to this cultural heritage. We need to get know thoroughly the history, the present state and the ongoing transformations of the medicinal plant world beyond the mainstreamed 20 species well, and pass on this diverse knowledge holistically with all the *pros* and *cons*. *In our reductionist haste we are at risk of losing many parts of the story, our grandmother’s story, the story of the ages, the philosophers, the scientists and the plants themselves* (Kingsbury, 2010). This alarming research and documentation gap need to be addressed holistically in a joint transdisciplinary effort. The well-established mostly grass-roots environmental education activities in South Tyrol are already fostering local environmental literacy for sustainable and healthy landscapes, but they need more governmental support such as an anchoring in the formal school curricula as key knowledge, like history, mathematics or languages. Medicinal plants are a cross-disciplinary object for learning, practising and participatory research. Our study demonstrates the key role of local communities in the linkage between medicinal herbs and nature conservation.

## Data Availability

The original contributions presented in the study are included in the article/Supplementary Material, further inquiries can be directed to the corresponding author.

## Footnotes

1 A systematic review on Scopus (October 2022) with the keyword combination (“medicinal” OR “herbal medicine”) AND “environmental education” revealed only twenty-five peer-reviewed publications mostly from environmental sciences, medicine, agricultural and biological sciences and social sciences since 1996 originating from 17 mostly non European countries with 9 studies from Brazil.

2 The processing of personal data was performed in accordance with the EU General Data Protection Regulation (EU-GDPR) and in compliance with the Berlin Data Protection Act (BlnDSG).

3 For characteristics of interviewees coded by I1, I2, I3 … to I14, the date and transcripts of the interview are documented in the Supplementary Material. Literal citations are given in italic letters.

4
www.kloster-neustift.it; www.schloss-goldrain.com; www.kraeuter-akademie.com

5
www.laimburg.it

6
www.winterschule-ulten.it

7
www.kräuterkraft.info

8
www.str-ka.it

9
https://www.pharmaziemuseum.it
