# Supplementary material for: Understanding old herbal secrets: The renaissance of traditional medicinal plants beyond the twenty classic species?
Source: Front Pharmacol. 2023 Mar 24;14:1141044. doi: 10.3389/fphar.2023.1141044 (PMC10079881; doi:10.3389/fphar.2023.1141044)
Supplement: Supplementary file 1 [file DataSheet1.docx]

Supplementary Material

Understanding old herbal secrets: the renaissance of traditional medicinal plants beyond the twenty classic species?

Marisa Milena Scherrer, Stefan Zerbe, Joshua Petelka, Ina Säumel

*** Correspondence:** Corresponding Author ina.saeumel@hu-berlin.de

The processing of personal data was performed in accordance with the EU General Data Protection Regulation (EU-GDPR) and in compliance with the Berlin Data Protection Act (BlnDSG). The anonymized transcripts are provided under the FAIR standards (findable, accessible, interoperable and reusable)^[[1]](#footnote-1)^ while ensuring protection of personal data.

# Characterization of Interviewees

| Stakeholder | Gender | Time in the topic (years) | Code | Duration of interview (min) |
| --- | --- | --- | --- | --- |
| Herbal Pedagogue | f | 10 | I1 | 20 |
| Herbal Farmer | m | 25 | I2 | 20 |
| Monastry | f | 30 | I3 | 40 |
| Herbal Blog | f | 10 | I4 | 25 |
| Natural Museum | m |  | I5 | 15 |
| Environmental Agency | f |  | I6 | 15 |
| Tourism Agency | f | 27 | I7 | 15 |
| Research Centre | m |  | I8 | 10 |
| Pharmacist | m | 15 | I9 | 30 |
| Herbal Farmer | m/f |  | I10 | 10 |
| Environmental NGO | f | 13 | I11 | 15 |
| Environmental NGO | f | 30 | I12 | 25 |
| SME | f |  | I13 | 15 |
| Herb Expert | m |  | I14 | 45 |

# Keywords

| *englisch* | *deutsch* |
| --- | --- |
| ecosystem restoration, nature conservation, nature protection, sustainability | Renaturierung, Naturschutz, Nachhaltigkeit |
| ethnobotany, ethnomedicine, herbal medicine | Ethnobotank; ethnobotanische Bedeutung, Heilpflanzen, Wildpflanzen, Speisepflanzen, Gesundheit, Heilkräuter |
| traditional medicine, traditional knowledge | Tradition, traditionelle Medizin, Brauchtum, alpine Heilkunde, Erfahrungsheilkunde, moderne Kräuterkunde |
| vascular plants, herbs, medicinal plant | Pflanzen: Gefäßpflanzen, Kräuter, Heilpflanzen, Speisepflanzen |
| cultural keystone species, culture, cultural importance indices, biocultural conservation, ethnobiology, conservation biology | Kultur: kulturelle Schlüsselarten, traditionelles und neues Wissen, (Allgemeinwissen), Knowhow, moderne Kräuterkunde, Natur, Regionalität, Nutzungsintensität, Vielfalt, Zeremonien, Legenden, Unersetzlichkeit, Verletzbarkeit, Brauchtum, Naturbrauchtum, Naturfest, Heilmethoden, Naturheilkunde |
| environmental education | Umweltbildung (Kinder- und Erwachsenenbildung: Kräuterpädagogik, Kräuterbauer, Kräuterwanderungen, Gesundheitsbildung, Umweltgedanke, Bewusstseinsbildung, Umweltschutz, Nachhaltigkeit, Schutz, Prävention |
| biocultural diversity, endangered plant species, conservation, ecological restoration, environmental ethics, human choice, land management, restoration ecology | biokulturelle Diversität: gefährdete Arten, Restorationsökologie Landmanagement, Umweltethik |

# Keywords assigned to questions

# How do you assess the interest and influence on traditional medicinal plants? Keywords: customs, empirical medicine, culture, folk medicine, traditional knowledge, ancient knowledge, alpine/modern medicine, know-how. Knowledge, ancient knowledge, alpine/modern medicine, knowhow

# Who do you consider relevant for the topic of traditional medicinal plants in South Tyrol? Keywords: awareness raising, experiential medicine, trad. Knowledge,

# Which South Tyrolean medicinal plant do you consider the most important for medicinal purposes? (nutrition, veterinary purposes, spiritual and cultic purposes, cosmetic and domestic) Keywords: alpine medicine, regionality, culture, ethnobotanical significance, customs, naturopathy, tradition

# Which South Tyrolean medicinal plant do you consider culturally most important in terms of intensity of use, ceremonies, legends, symbols, history, trade, irreplaceability? Keywords: cultural value, diversity, culture, legends, ceremonies, irreplaceability, healing effect, regionality

# To what extent are key species in South Tyrol formative for the preservation and restoration of ecosystems? Keywords: irreplaceability, indigenous plants, environmental protection

# Is there an increased focus on passing on knowledge about medicinal plants? What does the educational work look like? What role do medicinal plants play in environmental education? Do they play a role? How can more importance be attached to knowledge about medicinal plants? How relevant is the topic for children and young people today? How important is it to pass on knowledge to future generations? Keywords: environmental protection -> awareness raising, environmentalism, health education, nature conservation, environmental education, herbal education, adult education

# Transcripts of interviews

Person I1 (14.03.22)

*How do you assess the interest and influence on traditional medicinal plants?*

- existing interest; selected clientele

- Shift from older to younger audience

- Offer in constant change (herbal hike+cooking event) -> (multiple occupation with the herbs) -> better memorisation through experience

*Which South Tyrolean medicinal plant do you consider culturally most important in terms of intensity or variety of use, ceremonies, legends, symbols, history, trade and irreplaceability?*

- Top ten plants; many plants are not only medicinal plants but also edible plants at the same time

- Nettle as queen of the indigenous wild plants: important for nutrition, healing, clothing

- Tale of the seven swans (Hans Christian Andersen: dress of nettles) -> protective plant -> nettle is very defensive, makes strong at the same time -> master against all evil spirits (in former times the dead dressed with the plant)

- Nettle, dandelion, goutweed, daisies, ribwort (also very healing and tastes good), yarrow -> occur frequently (and are picked for healing teas or to eat etc.) -> no exploitation, damage to nature

To what extent do medicinal plants have a cultural value? (certain ceremonies, medicinal, symbolism)?

- Smoking of medicinal plants during the nights of incense

- Currently there is an upswing & interest in old traditions

- Offer changing; exchange with customers important (e.g. Walpurgis -> creation of a programme)

*What role do (cultural) medicinal plants play in environmental education? What does the educational work look like?*

- also on offer for schools/kindergartens, birthdays; nature conservation associations (herb treasure hunt) -> search, craft, eat

Primary school age: very interested (depending on parents' home -> garden etc.); teenagers: different interest, completely different topic (especially motivation important)

- Offer on request, especially for children and young people -> positive memory/experience creates awareness for the future

*Herbal education:*

- Knowledge transfer with fun (become active yourself and learn something)

- Focus on adult education, but also children's programme (on request by the school) -> Day to the tree, forest, meadow

- Orchard project by the State Office for Agriculture (LFL), mostly in autumn

*To what extent is it important to pass on knowledge to future generations?*

- School authority creates afternoon offers; nature conservation associations, horticultural associations, community (as part of the summer holiday programme)

- could be expanded (question of financing)

- What I know I can only protect and (not destroy)

Person I2 (14.03.22)

*How do you assess the interest and influence on traditional medicinal plants?*

- Interest seems to increase every year (environmental thought plays a very big role); people's longing for peace and recreation & nature is increasing more and more (renaissance of herbs all over Europe)

- Current topic (great interest); in the past there was a great tradition of herbs, mushrooms and animal substances -> but in the past people did not only work with herbs.

*Which South Tyrolean medicinal plant do you consider the most important for medicinal purposes?*

- Herbs that like harsh climates: masterwort; rosemary, marjoram, basil not native there

- Centuries ago, customs were still much more present

- On the Feast of the Assumption, certain herbs are picked (mullein, thyme, etc.) -> herb bunches are tied and consecrated in church.

- Dried herbs were burnt during thunderstorms and storms to ward off evil spirits.

- Rough nights (from 24 to 31 December or 5 January) -> herbs are smoked, nowadays: incense.

*What role do (cultural) medicinal plants play in environmental education (possibilities and limits)?*

- School classes visit farms -> contact also with herbs (active handling)

- Information on processing, history etc. -> Aim: arouse interest/awareness (learning about herbs is far too extensive); to be considered: Difference town & country

- Knowledge must be acquired over many years (long process)

- School classes: 1-2 mornings programme/lessons on the topic of "herbs" (very broad)

- Desire for longer-term project (spring: sowing etc. -> problematic: summer holidays in schools)

- on request: herbal cosmetics, production of herbal salt etc. (possibilities are endless)

- naturally native: yarrow, stinging nettle, St. John's wort, mountain fern, mullein, field thyme

*To what extent is it important to pass on knowledge to future generations?*

- very important; even better if research is done in this direction and old knowledge is made accessible

- Very little knowledge still exists (100 years ago: everything was done with herbs, especially women were very knowledgeable about herbs); much has been lost through conventional medicine

- Traditional customs are only present in fragments.

Person I3 (15.03.22)

*How would you rate the interest and influence on traditional medicinal plants?*

- Basic understanding of nature

- has always been a big concern (environmental protection and nature conservation) to bring into the classroom, then: Adult education

- South Tyrol has a well-established educational landscape and there are many free offers; good herbal educators/experts

- AHA experience is no longer achieved so often (but knowledge is relatively superficial)

-> in the courses: not much knowledge left

- great potential: unite the whole (reach all strata of the population -> senior citizens, children (also in schools), young people, adults

- early childhood: no money available, only plant care (school medicine was too expensive); do not go to the doctor when you have a cold

- South Tyrol (south; near Kalterer See) is not very big, but climatically very different; further north: Meisterwurz (alpine region)

- Elder was the farmer's pharmacy (no use of blossoms; no elderflower tea)

- still further south: quince (Hildegard von Bingen): Seeds as cough drops; compote has cooling effect -> for expectoration in rheumatic diseases due to diuretic effect;

- Mountain regions: Cranberry (strong fever-reducing)

- Calendula ointment (formerly with lard); today milking fat (full of preservatives)

- due to progress in orthodox medicine (and fast-moving times, also due to the woman's occupation and the child's compulsory schooling) -> antibiotics are administered quickly

- The word "health" was not allowed to be used (only the doctor's business).

- A few years ago: "Healthy through the year with herbs" -> "Vital through the year with herbs".

- in Italy the image of the alternative practitioner does not exist

- meanwhile in trend: great interest (problematic: either only orthodox medicine or not at all) -> use of herbs for prevention (to stay healthy), possibly also for pain relief

- Orthodox medicine is not alternative but complementary

*Which South Tyrolean medicinal plant do you consider the most important for medicinal purposes?*

- Elder: you have to take your hat off to elder and you have to kneel down to juniper (from the Alpine region)

- Incense is a real fad -> esoteric (originally old tradition)

- Elder needs our proximity (along the motorway everything is full of elder); can cope very well with different conditions -> strong medicinal plant (berries, blossoms; in former times also bark and roots)

- Sage (no ornamental gardens in former times) formerly in the garden: rosemary, wormwood

- Italians are inferior in knowledge about healing properties of plants, but are very grateful for information in this regard

- Wild herb cooking: learned mainly from Italians -> e.g. dandelion, hops (wild asparagus in Trentino), linseed (spinach plant)

*To what extent do medicinal plants have a cultural value? (certain ceremonies, medicinal, symbolism)?*

- first three daisies (said to protect against illness)

- Stinging nettle (for inflammatory diseases)

- Arnica (as a protective plant; wolf's eye) -> Arnica filled into a balloon bottle (stays there all year and turns brown) -> drops put into schnapps

-> in former times: at the edge of the field for protection

- Carrying a horse chestnut in the pocket of the trousers, because it protects against bad energies

*What role do (cultural) medicinal plants play in environmental education?*

- accelerated by bee mortality, more present in the media

- more and more marginal plantings with natural meadows

*What does educational work with medicinal plants look like? (institutions?)*

- Fun factor very important

- Awareness/sensitivity has become stronger (but also very dependent on the parental home)

- Practical work is important (quotation from Goethe: "It is not enough to know, one must also apply it. It is not enough to want to do it, one must also do it).

- today: mission and theme for the present generations (crises must always be seen in a positive light)

- Herbs for health maintenance and joy of life (" This is my health")

- thoughtless consumption has decreased (forced by Corona); urban gardening shows that people need to get involved

- "It's not the quantity that matters, but the harmonies".

- Offers are very well received by children

- Youth organisation/centre (Jump in St. Michael Eppan) -> have created a garden; in summer there are projects (one week: we herb witches etc.)

- A lot is also being done in teacher training (more support from the state is needed).

Person I4 (18.03.22)

*How do you assess the interest and influence on traditional medicinal plants?*

- South Tyrol at the top, strongly increasing in recent years.

- Medicinal herbs are still deeply rooted, especially in South Tyrol; gap of knowledge (not exact anymore) -> basic understanding, need exists

- South Tyrol (rich in plants), abundance of Mediterranean species (always significant)

-> close attachment to herbal healing

- Increasing gaps due to changed living conditions: close living together and close exchange of such knowledge is no longer given

- healing emphases in remote valleys are coming back into play and are now playing a greater role again (many young people who no longer have knowledge) -> strong increase

- very, very deep scepticism towards modern medicines (aversion to synthetic medicines), health system in South Tyrol, treatment doctor and patient (no longer partnership, but determining) ; are there no alternatives? What did our ancestors use?

- Recourse to home remedies to the point of exaggeration

- Deep faith,trust (something God-given, indispensable help) in the power of plants (different from many other regions)

- Older generations: Arrested in knowledge (no enquiries as to why etc.), circulation of a lot of false information

- younger generation: enlightening knowledge (why does a plant help?); vulnerability noticeable

- great interest among women as soon as they want to have children or after childbirth; more intensive demand for medical knowledge.

*Who do you consider relevant to the topic of traditional medicinal plants in South Tyrol?*

- Interest is leading

- a kind of globalisation is taking place

*Which South Tyrolean medicinal plant do you consider the most important for medicinal purposes?*

- Dandelion and yarrow

- Dandelion: plays an important role in everyday nutrition (cleansing, liver plant)

- Yarrow (gynaecology)

- 10. rule/day (all plants that are not protected)

- 15 freely collectable plants (yarrow, nettle, elder)

- More special, alpine: masterwort (from higher altitudes); musk yarrow (grandpa made schnapps from it)

- Arnica (one of the top plants); is under nature protection and may not be picked, often contaminated by boring flies (partly unusable by now)

- Arnica is collected by Italians (travel there specially -> sold on local markets in southern Italy, exploitation) -> local population very sensitive about it

*Which South Tyrolean medicinal plant do you consider culturally most important in terms of*

intensity or variety of use, ceremonies, legends, symbols, history, trade and irreplaceability?

- Bed. Training of herb farmers with an approved farm (only then may they sell) -> Protection of herb farmers

- Medicinal plants have migrated strongly into cultivable plants: marigold, lemon balm, peppermint

- Native plants rather in the background except for a few (cultural anchoring with stories -> many plants deeply anchored in Ladin legends -> more likely to be known by older people)

Shift towards cultivated plants (there is a lack of knowledge, stories etc., almost non-existent)

- very different in different places and families (e.g. Ascension bushes: palm bushes, Easter plants that have healing power (spiritual); harvest thanksgiving, Rauhnächte (incense has high value) -> specialists who have rebuilt knowledge and retell it with great success (also touristically)

- Restrictions are very strict (for herbal hikes): not above 500 m, if no mountain guide training available -> is exploited via tourism offices

- E.g. cook training: other evaluation and estimation by the cook (speciality of the plants -> unique selling proposition; a business oriented towards a business: culinary, well-being, application up to deep medicine

*To what extent are key species in South Tyrol formative for the preservation or restoration of ecosystems?*

- little can be achieved with bans, but through awareness (I value what I know, I protect what I value)

- Promote nature conservation much more (if meadow is fertilised, arnica is gone -> understood by most)

- Most people are looking for a way out (they feel too weak to make a difference) -> small steps help.

- Goal: understanding in the population why money has to go into nature conservation (many actions have left traces because plants are important for our health and treatment; was relatively unimportant for a while)

- Courses filled up very quickly even after Corona restrictions were relaxed

- Alpine forest bathing -> larch pitch ointment (everyone in South Tyrol must have it; miracle things are said about ointment)

- Mistrust: Are they allowed to do this? Where do authorisations come from? uncritical acceptance)

- optional for all (broad-based education): FNL (Friends of Natural Living - Austrian association) - free to all (broad education): FNL (Friends of Natural Living - Austrian association); winter school; evenings in the village libraries (large educational institutions -> are very active, disseminate knowledge, very small-scale).

*How important is it to pass on knowledge to future generations?*

- it lives from this; it has always been like this (primal knowledge that counts as folk knowledge)

- living knowledge -> from person to person most important (sensitisation in the family -> difficult to achieve in school -> adult education: what is right, what is applicable? Where are limits?

- Children can be taught how important daisies etc. are (first contact through family etc. -> holiday programme etc.)

- Village communities still have a different value (unimaginable that someone does not know that daisies cannot be eaten -> taken for granted)

- Attention: Social media (multi-layered to be considered in the future

Person I5 (21.03.22)

*How do you estimate the interest and influence on traditional medicinal plants?*

- in the province: many courses also from the adult education centres (also from the Bildungshaus Kloster Neustift, Bildungshaus in Meran - Urania).

- medicinal herbs have a great tradition

- great knowledge, much is done in the private sphere

- new phenomenon: between hype, esotericism and real discussion/use of medicinal plants

- People sneak in, get know-how -> no fan of traditional history; some earn a lot from courses on medicinal herbs

- Statements: "plants are only for people" -> many different people

- naive: last stocks of centaury are still being tried to be found (nature conservation -> not comprehensible: Use of rare, endangered species for dubious application of medicinal plants)

- not for nothing requirements in pharmacology: plants have to be cultivated in order to be sure that the ingredients are available in sufficient quantities to have an effect (broad field of discussion).

*Which South Tyrolean medicinal plant do you consider the most important for medicinal purposes?*

- Traditional knowledge vs. new knowledge/hype (is not congruent)

- There is a big difference between the experienced knowledge of a 90-year-old and the knowledge of a 30-year-olds who read information from books.

*To what extent are key species in South Tyrol formative for the conservation or restoration of ecosystems?*

- is contrary to each other: last protected areas are damaged

- extensive meadows with arnica are promoted -> promotion of the use of arnica (aspect is not a priority)

- Nature Conservation Act: partially protected plants (e.g. arnica -> extra case: FFH species) to avoid overexploitation

- Collecting permit if necessary (purpose must be stated -> coordinated thing that works)

- Protection of medicinal herbs unequal to ecosystem services

*What role do (cultural) medicinal plants play in environmental education?*

- Deepen knowledge of flora (real knowledge of species, which is rapidly being lost), because this is hardly promoted (no more publications)

- Taxonomy is constantly changing (not frozen)

- mainly flora and ecology; application is not an issue

- NATURE CONSERVATION in the first place

- out of many hundreds of plant species traded as medicinal herbs, effects can only be proven in 10 (without belief in self-healing etc.)

Person I6 (21.03.22)

*How would you rate the interest and influence on traditional medicinal plants?*

- mixed: partly very high vs. not at all significant

- don't know if it has become more or less (not greatly displaced by orthodox medicine)

- no special medicinal plants, except regionally typical due to the climate

- Legends, symbolism etc.: no significance

*What role do (cultural) medicinal plants play in environmental education?*

- Nothing about medicinal plants or plants in general

- Life in water, biology: tiger mosquitoes, prevention measures

- general: from noise to paper

Person I7 (22.03.22)

*How do you assess the interest and influence on traditional medicinal plants?*

- South Tyrol tourism: territoriality (local, also plants), authenticity (closely linked to territory), sustainability on everyone's lips.

- Medicinal plants grow locally (integration in food, medicine) -> depending on territory

- Trend: wild herbs (partly also medicinal plants); old knowledge that has been lost is tried to be reactivated.

- Ignorance: something is uprooted because there is no awareness -> Knowledge: completely different reaction (very positive)

- Nature and regionality is interesting and gives confidence also for the future (earlier times can be better understood -> tradition)

- Health trends and prevention -> natural remedies

- since a few years wild herbs are rediscovered (also in local gastronomy)

-> wild herb weeks (April, May); medicinal plants already more specific

- Major role in environmental education: guided tours all year round, but especially in spring on wild herbs (overlapping with medicinal plants)

- From a touristic point of view: as broad an offer as possible (also for the ignorant) General knowledge (taste is also very important, how can I cook with the plants at home, etc.)

- Offer geared to adults and possibly families (little change in recent years)

- Festivals associated with pagan cults: Incense burning at the turn of the summer night -> at the same time explanation of the tradition that has been handed down.

Person I8 (22.03.22)

*How do you assess the interest and influence on traditional medicinal plants?*

- exists and is becoming stronger; big trend: herbal walks (herbal educators)

- Target group unclear (partly also younger people, not only adults)

*Which South Tyrolean medicinal plant do you consider the most important for medicinal purposes?*

- 45 Growers: herb cultivation, drying, processing and marketing

- Lemon balm, mint (main ingredient in tea blends), sage, rosemary, basil, flowers: cornflower, camomile, calendula

- Wild collection: some, such as arnica (widespread) -> arnica schnapps for rubbing in joint complaints; yellow gentian (gentian schnapps at the huts in Tyrol).

*To what extent are key species in South Tyrol for the preservation or restoration of ecosystems?*

- In the population, the belief that wild collected material has a better effect; the rarer the better

- Pressure on nature (knowledge is partly not available about cultivable plants?)

- Arnica (cultivated varieties): which are better in terms of ingredients

- always dependent on cultivation conditions

- nothing was wiped out by collecting wild plants etc.

- problematic: centaury, which is collected

Person I9 (24.03.22)

*How do you assess the interest and influence on traditional medicinal plants?*

- Interest is there; traditional empirical medicine is only present in traces; mainstream plants are used by most users

- there is a lack of old traditional things that were common practice 100 years ago -> heterogeneity of herbal medicine is no longer present today

- in former times: from valleys and valleys already very different; today compared to larger geographical areas: Central Germany and Northern Italy no big differences (herbal books are available)

- Reduction to few medicinal plants (20 that are used); earlier: 400 medicinal plants -> not much left of unknown medicinal plants

- Arnica, for example, may be harvested for personal use (regulations vary greatly) > Nature conservation prevents the harvesting of e.g. edelweiss for stomach aches (not cultivated)

*Which South Tyrolean medicinal plant do you consider the most important for medicinal purposes?*

- modern herbalism: arnica, mountain pine, juniper, comfrey (alpine)

- Empirical medicine (still some plants that are no longer used in modern herbalism): Masterwort, -> panacea; bloodroot, yarrow, lady's mantle (experiential medicine).

Which South Tyrolean medicinal plant do you consider culturally most important in terms of intensity or variety of use, ceremonies, legends, symbols, history, trade and irreplaceability?

- in former times much more anchored (many stories)

- Willow (willow catkins) ->Collected on Sundays, consecrated in church, kept in the parlour; today: burned during approaching thunderstorms; in former times also used more for folk medicine: a few willow catkins eaten in spring to be protected against sore throats and colds all year round.

- 2nd plant that is still very much used culturally (partly in South Tyrol; towards the main Alpine ridge): Masterwort for incense at Christmas -> instead of incense.

- Raunächte with masterwort in South Tyrol; Maundy Thursday soup

- 30igen (women's) bouquet is put together on 15 August and is used in a similar way to willow catkins (thunderstorm prophylaxis or if someone is ill, herbs are smoked; in former times: mainly women's herbs ( bedding on fragrant herbs)

-> today only consecrated in the church

- Collecting between 15 August and 8 September (women's days); Assumption of Mary

*To what extent are key species in South Tyrol for the preservation or restoration of ecosystems?*

- Collecting and using medicinal plants is closely linked to intact agriculture

- Problem: fertilised meadows in South Tyrol; sparsely available areas lead to the extinction of medicinal plants (treatment with pesticides etc.)

- Medicinal plant use closely related; some disappeared due to fertilisation of meadows: Horseweed, Centaury, Arnica (intermediate areas that are fertilised or alpine meadows that are developed and / or manure applied).

- major problem (more so than in other regions of the Alpine area)

*What role do (cultural) medicinal plants play in environmental education (possibilities and limits)? Do they play a role at all?*

- plays a role; with own children: Medicinal plants very good access for environmental education for children: cultural links are common (many books and stories, legends, explanations) -> there is no medicinal plant for which there is no story.

- stories that sound exciting to children -> medicinal plants have stories

- central for environmental education; things stay in the mind; few plants that cannot be used at all

*How can (more) importance be attached to knowledge about medicinal plants in environmental education?*

- Little difference between town and country

- Children do not know many names (starts with deciduous trees etc.)

- Health education belongs much more in schools (medicinal plants fit well -> possibility for self-help for mild or moderate complaints -> lift over general health education

- Environmental education: many synergies

Person I10 (29.03.22)

*How would you rate the interest and influence on traditional medicinal plants?*

- Very high; in the last few years it has increased strongly.

Who do you consider relevant to the topic of traditional medicinal plants in South Tyrol?

- Heinrich Abraham, certified herbalist, and Martha Mulser and South Tyrolean herb growers.

*Which South Tyrolean medicinal plant do you consider to be the most important for medicinal purposes?*

- Nettle, yarrow, marigold (the three most important).

To what extent are key species in South Tyrol for the preservation or restoration of ecosystems?

- Very important

*What role do (cultural) medicinal plants play in environmental education and what does educational work with medicinal plants look like?*

- At the moment still far too little

- In development; interest is growing

*To what extent is it important to pass on knowledge to future generations?*

- Old knowledge must be preserved

Person I11 (30.03.22)

*How do you assess the interest and influence on traditional medicinal plants?*

- Aim: To arouse interest and joy in nature and, using the example of the "diversity of medicinal plants and their use for one's own "well-being", also to influence one's behaviour towards nature -> learning to appreciate and respect nature

- Increasing interest in the last years (no information to what extent knowledge is also applied or deepened -> many courses and trainings

- There is a danger that more and more plants will be taken from nature -> current regulations regarding the ban on picking and uprooting in protected areas (nature parks, biotopes) must be observed.

Who do you consider relevant to the issue of traditional medicinal plants in South Tyrol?

- Laimburg (herb cultivation - not only local ones - for farmers' wives and interested people); Neustift Monastery (herb teacher training); Winter School Ulten ( 3-year training in Alpine herbology); Sigrid Thaler (head of FNL herb academy in South Tyrol); FNL= Freunde naturgemäßer Lebensweise; Gottfried Hochgruber (health consultations - alternative healing methods); various herb women who offer courses (e.g. Dora Somvi, Hil Hildegard, Dora S.). e.g. Dora Somvi, Hildegard Kreiter); kräuterkraft blog of the South Tyrolean herb women.

*Which South Tyrolean medicinal plant do you consider the most important for medicinal purposes?*

- Not " The most important"; certainly several that are used: St. John's wort, arnica, camomile, quendel, comfrey, birch, masterwort, mountain pine (essential oil), gentian.

- Personal and need-oriented use

*Which South Tyrolean medicinal plant do you consider culturally most important in terms of intensity of use or diversity, ceremonies, legends, etc.?*

- Intensity of use or variety: camomile, dandelion, stinging nettle, yarrow, lady's mantle, valerian, dog rose (fruits rose hips), linden, elderberry.

- Ceremonies: Herbs for incense, juniper

- Herb consecrations on 15 August- herb bushes: Mullein, St. John's wort, centaury, yarrow, chamomile, wormwood, valerian, (peppermint) and arnica are part of the "nine".

To what extent are keystone species in South Tyrol for the preservation or restoration of ecosystems?

- Medicinal herbs are not decisive in the restoration of wetlands

*What role do (cultural) medicinal plants play in environmental education?*

- no knowledge about schools

- in herb gardens guided tours for adults and children, children's afternoons, courses: Worth knowing: recipes and practical information on processing herbs: tea mixtures, oil extracts, tinctures, cosmetics (ointments, creams), scented mixtures; use of herbs in cooking

- about the diversity in nature using the example of medicinal plants and their use for one's own well-being also to influence one's behaviour towards nature: Respect and appreciation of nature

*To what extent is it important to pass on knowledge to future generations?*

- very important, so that knowledge is not lost

- With appropriate knowledge one is more independent of conventional medicine

- Responsibility is taken for one's own health; strengthening of one's own cultural identity

- Through this knowledge, one learns to appreciate and respect nature.

Person I12 (04.04.22)

*How do you assess the interest and influence on traditional medicinal plants?*

- Very much increasing; was not so current a few decades ago, but since approx.

10-15 years the interest in South Tyrol has been growing strongly (Increasing every year)

Interest in medicine, herbology -> have parents who have also been interested in this (50+)

*Who do you consider relevant for the topic of traditional medicinal plants in South Tyrol?*

- Regarding training places, a lot has happened in the last 10 years (15 years ago hardly any courses or trainings were possible)

- Educational institutions: Neustift Monastery, Goldrain Castle in Vinschgau, Herbal Academy (hundreds of people are trained as herbal experts) -> pass on knowledge at various workshops, seminars etc.

- Gastronomy has risen to the top (30 years ago there was no interest in herb walks etc.) -> today there are hardly any major hotels that do not have this in their (weekly) programme: needs a lot of skilled workers.

*Which South Tyrolean medicinal plant do you consider the most important for medicinal purposes?*

- difficult to break it down to a few

- medicine cabinet (everyone has ointments at home): Larch resin ointment -> ointment against abscesses, splinters, strong warming against tension, resin is strong expectorant -> bronchial ointment; calendula and arnica ointment.

- Tinctures: Iceland moss tincture for flu-like infections (very important in tradition), masterwort, angelica (more for incense -> incense is very topical: mugwort, juniper, spruce resin, angelica).

*Which South Tyrolean medicinal plant do you consider culturally most important in terms of intensity or variety of use, ceremonies, legends, symbols, history, trade and irreplaceability?*

- Customs not necessarily linked to churches -> more and more are turning away, feel out of place

- CUSTOMS must not be forgotten

- Cycle of the year festivals, nature customs -> church festivals have resulted from this

- Christmas: Birth of the light -> from day to day it becomes brighter again, incense of plants

- Easter: resurrection of nature (where did the church get this from?)

- Assumption: festival of reapers? Giving thanks that the herbs have been harvested

- every church festival is a nature festival (one does not exclude the other: trad. vs.

thanks to nature)

*To what extent are key species in South Tyrol for the preservation or restoration of ecosystems?*

- Boom in recent years: herbal educators passing on knowledge;

- Question about the extent to which there is too much interference with nature? Nettles etc. -> stress on ecosystems?

- Contradiction

1) Awareness of nature is learned: collect in such a way that one does not notice that one has collected.

2) Nature provides us with enough (no fear that something will be destroyed), rather destroyed by conventional cultivation; supermarket: storage, transport, packaging must be taken into account -> greater damage?

- People have to learn that conscious collecting is not a problem, but the packaged is the problem

- No burden on nature (those who know nature, love nature and appreciate it)

Environmental education- increased focus on passing on knowledge about medicinal plants? What does the educational work look like?

- each training in South Tyrol is different -> each has a different focus

- Herb expert training: getting to know nature (400 different plants: alpine plants, garden herbs, wild herbs, neophytes, poisonous plants)

- Getting to know nature without judgement (poisonous plant just as important and beautiful and significant as medicinal herb in the garden etc.) -> no weeds either

- Neophytes also have a significance, perhaps important bee plants etc.

- ⅔ of outdoor education in different locations.

- ⅓ in the room: tinctures, ointments, chemotherapy, customs and incense, aromatherapy, homeopathy, Bach flowers -> many doors are opened, immersion takes place in further targeted courses.

- How can I use the power of nature and what possibilities do I have?

- Course participants are very wide-ranging in terms of age (from 20-70)

- also professional (from farmers to academics etc.); many educators (naturopathy in the profession); some from nursing professions (alternative medicines, wraps, washing etc.) -> all professions represented

- A small part of the participants use their knowledge professionally, but most of them use their knowledge privately in the family (many young mothers).

*How can (more) importance be attached to knowledge about medicinal plants in environmental education?*

- difficult to answer, because political knowledge is missing

- more and more people are interested in nature -> still a long way to go (last 2 years: back by decades)

Person I13 (04.04.22)

*How do you assess the interest and influence on traditional medicinal plants?*

- meanwhile stronger again

- in 80/90-ies: less importance -> everything from Far East; natural medicine was rather in the background

- Return to region, which is good; also in hotels understood that applications with local plants work

*Who do you consider relevant for the topic of traditional medicinal plants in South Tyrol?*

- In the past there have always been herbal men and women

- Herbalists in the region: nobody knew exactly what they were doing (personal healing powers, also ointment)

- Kneipp (not from there), always different people: e.g. Sigrid Thaler, who is very much in demand and gives many courses (herbal medicine)

*Which South Tyrolean medicinal plant do you consider the most important for medicinal purposes?*

- Arnica (under nature protection) -> everyone knows schnapps

- St. John's oil is just as well known (flowers in oil and then used for burns)

- Well-known and valuable plants that everyone knows

- Calendula (used to be used with lard) -> pioneer plants

- Camomile, stinging nettle (very, very valuable medicinal plants -> powers are underestimated)

*Which South Tyrolean medicinal plant do you consider culturally most important in terms of intensity or variety of use, ceremonies, legends, symbols, history, trade and irreplaceability?*

- 15 August Assumption of the Virgin Mary: herb bouquet carried into church for blessing (herb bouquet: mullein, raspberry leaves, etc.).

- The bouquet is hung up and during a thunderstorm a piece is taken off and burned-> so that there is room for something new.

-> herbalists still do this

*To what extent are key species in South Tyrol for the preservation or restoration of ecosystems?*

- of course medicinal plants are important: plants that grow wild on site have greater value than plants that are sown -> plant chooses its place and grows as it grows (is not controlled by humans)

- Sigrid Thaler makes trainings and lectures

- too little is done and promoted by politics

- People are interested, but there is too little political support.

*How relevant is the topic for children and young people today?*

- Very important, should happen especially in schools.

- few have knowledge at home (even less in the city than in the country)

- much more be accommodated in school) -> many no longer have a connection to nature

- many lack the opportunity (is frightening and a pity)

Person I14 (14.04.22)

*How do you assess the interest and influence on traditional medicinal plants?*

- deals a lot with medicine, but also (medicinal) herbs;

- Healing: to heal you have to be sick first; wrong approach (nature has never done anything bad to man, it only gives something to man)

- 5 plants are enough for "healthy" life (change according to phase of life)

- plant cannot heal (goal: plant gives the power/energy for change)

- not one is bad and the other good -> choice of remedies

- instead of medication (take one substance over and over again), plants -> energy/power: changes thinking and acting (learn to understand people)

- different way of dealing with nature (people can change things with knowledge -> see nature differently as a result)

- Culture is knowledge that has been handed down (old+new knowledge -> passed on to the next generation); new knowledge can be built on old knowledge.

- New alternative healing methods (build on the foundation of old knowledge)

- today: German name of the plant I understand must be learned; ex.: Meisterwurz: master of all roots (highest good/highest form of plants in the Alps)

- Yarrow; in former times 1st animal husbandry (tragedy when animal died; nutrition of man) -> name yarrow originated in the mountains; sheep eat yarrow only when they are sick (bundle of the plant -> hung in the barn to dry and dried for the winter when sheep were sick)

- German names are significant for relation to the plant

- Understanding ancient knowledge and applying/adapting it to today (highest good: passing on knowledge); change only takes place through increase in knowledge

- There are only plants, no medicinal plants Nature must not be judged (see, touch, smell & learn social behaviour and respect towards nature: nature feeds us, not industry)

1. https://www.go-fair.org/fair-principles/ [↑](#footnote-ref-1)
